# Monocytes, Macrophages, and Metabolic Disease in Atherosclerosis

**DOI:** 10.3389/fphar.2019.00666

**Published:** 2019-06-13

**Authors:** Michelle C. Flynn, Gerard Pernes, Man Kit Sam Lee, Prabhakara R. Nagareddy, Andrew J. Murphy

**Affiliations:** ^1^Haematopoiesis and Leukocyte Biology, Baker Heart and Diabetes Institute, Melbourne, VIC, Australia; ^2^Department of Nutrition Sciences, University of Alabama at Birmingham, Birmingham, AL, United States

**Keywords:** diabetes, obesity, monocyte, macrophage, atherosclerosis

## Abstract

Atherosclerotic cardiovascular disease (CVD) is a lipid-driven chronic inflammatory disease, in which macrophages are responsible for taking up these lipids and driving disease progression. Over the years, we and others have uncovered key pathways that regulate macrophage number/function and identified how metabolic disorders such as diabetes and obesity, which are common risk factors for CVD, exacerbate these pathways. This ultimately accelerates the progression of atherosclerosis and hinders atherosclerotic regression. In this review, we discuss the different types of macrophages, from monocyte-derived macrophages, local macrophage proliferation, to macrophage-like vascular smooth muscle cells, that contribute to atherosclerosis as well as myeloid-derived suppressor cells that may have anti-atherogenic effects. We will also discuss how diabetes and obesity influence plaque macrophage accumulation and monocyte production (myelopoiesis) to promote atherogenesis as well as an exciting therapeutic target, S100A8/A9, which mediates myelopoiesis in response to both diabetes and obesity, shown to be effective in reducing atherosclerosis in pre-clinical models of diabetes.

## Introduction

Cardiovascular disease (CVD) remains the leading cause of death worldwide and is of major concern in populations with increasing prevalence of metabolic disease (World Health Organization, 2017). Diabetes and obesity are both independent risk factors for CVD, with diabetic and pre-diabetic patients accounting for 65% of all CVD deaths (Barr et al., 2007; Flint et al., 2010). Complicating the situation further, obesity-associated diabetes accounts for 90–95% of adult diabetes diagnoses (Mozaffarian et al., 2015). Importantly, traditional risk factors, such as dyslipidemia and hypertension, fail to account for the increased risk of CVD in diabetes. Moreover, through the JAPAN-ACS trial, it was found that, while statin therapy is equally effective in lowering cholesterol levels in patients with and without diabetes, patients with diabetes exhibited impaired plaque regression (Hiro et al., 2010).

In recent years, targeting inflammation in CVD with the Canakinumab Anti-inflammatory Thrombosis Outcome Study (CANTOS) demonstrated that inhibiting interleukin-1β (IL-1β) reduces the incidence of secondary cardiovascular events, independent of lipid lowering (Ridker et al., 2017). Further, it has become well established through pre-clinical models that changes in the inflammatory milieu of atherosclerotic plaques, as well as systemic inflammation, play an important role in the development and vulnerability of atherosclerotic plaques to rupture. In particular, macrophages and their circulating precursors, monocytes, have been shown to play an important role in the pathogenesis of atherosclerosis (Murphy and Tall, 2016). This review will discuss the role of different sources of macrophages in atherosclerosis in the context of diabetes and obesity with a focus on the role of monocyte-derived macrophages and myelopoiesis in promoting atherosclerosis.

## Macrophages in Atherosclerosis

Atherosclerosis is primarily driven by the combination of lipid accumulation and immune cells within the plaque (Moore et al., 2013). In particular, macrophages play a crucial role in the development of atherosclerosis through uptake of modified cholesterol, in particular oxidized low-density lipoproteins (oxLDL), and the subsequent impairment of cholesterol efferocytosis, resulting in lipid-laden macrophages known as foam cells. These foam cells have impaired migratory capacity and thus become trapped within the plaque where they die and form a necrotic core (Pagler et al., 2011). In mice, macrophages are particularly important in promoting the development of early atherosclerotic lesions beginning to accumulate during the formation of fatty streaks; however, they are also involved in the transformation to a mature, inflammatory and unstable lesion, vulnerable to rupture (Moore and Tabas, 2011). In humans, plaque development begins at sites of disturbed flow through the formation of vascular smooth muscle cell (VSMC)-rich adaptive intimal thickening and the retention of modified lipoproteins, which precede immune cell infiltration (Otsuka et al., 2015). Macrophages subsequently accumulate within the lesion and increase throughout disease progression (Otsuka et al., 2015).

**Figure 1:**
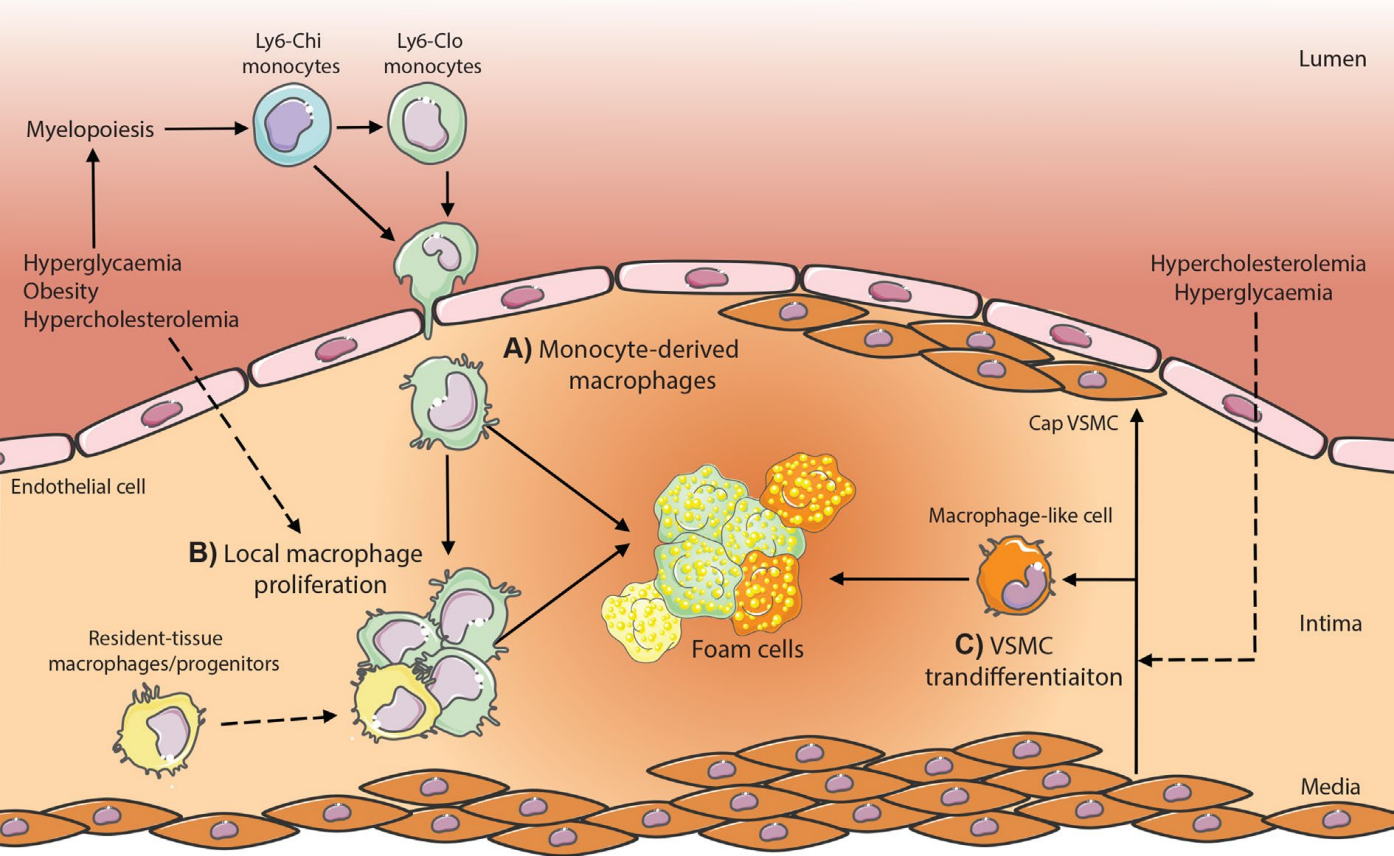
Potential contributing macrophage sources in atherosclerotic plaques in metabolic disease. **(A)** Monocyte-derived macrophages are produced through enhanced myelopoiesis in response to hyperglycemia, hypercholesterolemia, or obesity-associated adipose inflammation and infiltrate the plaque where lipid-loading triggers transformation into foam cells. **(B)** Local macrophage proliferation and **(C)** vascular smooth muscle cell (VSMC) transdifferentiation within the plaque contributes also have the potential to produce foam cells; however, whether metabolic dysregulation (hypercholesteremia, hyperglycemia) modulates these processes is yet to be established *in vivo*.

During the early fatty-streak stages of atherosclerosis in mice, M2 (pro-resolving) macrophages predominate in the lesion, whereas more advanced plaques are suggested to exhibit a shift towards an M1 (inflammatory)-dominant state (Khallou-Laschet et al., 2010). However, during plaque regression, decreases in macrophage content is accompanied by the restoration of the M2 phenotype (Rahman et al., 2017). In humans, macrophages found to express M1 or M2 markers have both been identified in the lesion, with M1 macrophages predominate within the rupture-prone shoulder regions of the plaque (Stoger et al., 2012). In vulnerable, symptomatic plaques, markers of M1 macrophages (CD68, CD11c) are increased while M2 markers (CD163 and mannose receptor) are decreased (Cho et al., 2013). Furthermore, a myriad of macrophage subtypes and species-specific populations have been defined in both mice and humans and reviewed elsewhere (Bobryshev et al., 2016), all contributing to different roles in atherosclerosis. In diabetic models, hyperglycemia significantly increases the abundance in plaque macrophages, thereby inducing a twofold effect: 1) accelerating plaque formation and 2) hindering plaque regression, even with adequately controlled plasma cholesterol levels. This increase in plaque macrophages is primarily caused by a significantly higher proportion of circulating monocytes entering the atherosclerotic lesion (Parathath et al., 2011; Nagareddy et al., 2013; Distel et al., 2014). However, alternative sources of macrophages may also contribute to lesion development, which are discussed below and summarized in **Table 1** and **Figure 1**.

**Table 1:** Summary of the sources of macrophages and macrophage-like cells within the plaque and their role in atherosclerosis and metabolic disease.

Macrophage type	Resident tissue macrophages	VSMC-derived macrophage-like cells	Monocyte-derived macrophages	Monocytic MDSCs
*Origin*	Likely to ultimately originate from monocytes	Vascular smooth muscle cells	Bone marrow stem cells *via* medullary or extramedullary (spleen) myelopoiesis	Bone marrow stem cells *via* medullary or possibly extramedullary (spleen) myelopoiesis
*Subsets and markers*	CD68, Mac-2 Brdu^+^ following labeling	CD68, Mac-2	CD68, Mac-2 will incorporate Brdu	
			**Monocytes**	
			Mouse: CD11b^+^Ly6G^-^ and Ly6C^hi^ or Ly6C^lo^	Mouse: CD11b^+^Ly6G^-^Ly6C^hi^ Also lack CD11c and MHC class II
			Human: HLA-DR+ and CD14^++^CD16^−^, CD14^+^CD16^+^ or CD14^dim^CD16^+^	Human: CD14^+^HLA-DR^-/lo^
*Limitations of markers*	Due to shared markers, lineage tracing studies still required to confirm contribution of these cells	Due to shared markers, lineage tracing studies required to confirm contribution in metabolic diseases	Some studies lack sufficient evidence to rule out potential contributions of macrophage from other sources. Bone marrow transplant studies and monocyte labeling provide good evidence for their contribution.	Most studies in mice fail to discriminate MDSCs from monocytes and/or neutrophils
*Function*	Pro-atherogenic	Pro-atherogenic Reduced phagocytic capacity	Pro-atherogenic	Anti-atherogenic? Immunosuppressive
*Mouse vs. human*	Minimal proliferation of macrophages identified in humans, unknown macrophage origin	Evidence of co-expression of SMC and macrophage markers. May contribute to macrophage content identified by immunohistochemistry.	Circulating monocytes correlate with disease	Associated with ACS in humans Role in mice unclear
*Affected by diabetes/obesity*	↑ total plaque macrophage content in diabetic mice Unknown if from resident tissue macrophages	Promoted by hypercholesterolemia and hyperglycemia (*in vitro*), unknown in models of diabetes/obesity	↑ in circulation in diabetes/obesity ↑ lesion entry in diabetes ↓ lesion egress in diabetes	↑ in circulation in T1D patients ↑ in obese adipose tissue Not known if altered in the lesion

VSMC, vascular smooth muscle cell; MDSC, myeloid-derived suppressor cell.

## Monocyte-Derived Macrophages

Elevated circulating leukocytes are associated with increased CVD risk, with this association primarily driven by monocytes and neutrophils (Olivares et al., 1993; Danesh et al., 1998; Orchard et al., 2003; Coller, 2005). Monocyte abundance is an independent risk factor for CVD, with monocytosis causally linked to both the acceleration of atherosclerotic lesion progression and impaired lesion regression (Swirski et al., 2007; Tacke et al., 2007; Waterhouse et al., 2008; Nagareddy et al., 2013). In atherosclerosis, plaque macrophages are largely derived from circulating monocytes infiltrating the plaque. Endothelial activation induces the arrest of monocytes onto the vessel wall where they transmigrate into the arterial wall, maturing into macrophages. Adoptive transfer of GFP-labeled monocytes into 10-, 20-, and 50-week-old spontaneously atherosclerotic *Apoe* *^−/−^* mice shows that the degree of monocyte infiltration is increased throughout disease progression (Swirski et al., 2006). Moreover, impaired monocyte trafficking to plaque sites attenuates atherogenesis, as demonstrated in mice lacking either the monocyte chemotactic protein 1 (MCP-1) or its receptor CCR2, which strongly supports the hypothesis that monocyte infiltration is required for atherogenesis (Boring et al., 1998; Gosling et al., 1999). A seminal study by the Mallat group further demonstrated the importance of monocyte-derived macrophages *via* combined inhibition of CCL2 alongside CX3CR1 and CCR5, which are also required for monocyte trafficking, resulting in a 90% reduction in atherosclerosis in *Apoe* *^−/−^* mice correlating with levels of circulating macrophages (Combadiere et al., 2008). In mice, monocytes have been shown to enter into the plaque more readily in the context of diabetes and hypercholesterolemia (Swirski et al., 2007; Tacke et al., 2007; Murphy et al., 2011; Parathath et al., 2011; Nagareddy et al., 2013). Direct evidence of monocyte infiltration through tracking studies showed that monocyte entry is increased in diabetes while simultaneously impairing egress of these cells out of the plaque, resulting in overall increased retention (Parathath et al., 2011; Nagareddy et al., 2013). This corresponds to an increase in macrophage content in the plaques of both diabetic mice and humans (Moreno et al., 2000; Parathath et al., 2011; Nagareddy et al., 2013).

## Resident-Tissue Macrophages

While macrophages have traditionally been considered to be derived from bone marrow (BM) monocytes, evidence in a number of organs has demonstrated that, under steady-state conditions, monocyte-derived macrophages contribute little to overall macrophage populations (Yona et al., 2013). Myeloid cells, including macrophages, are known to originate from two stages of hematopoiesis during development: 1) primitive hematopoiesis, occurring in the yolk-sac prenatally, and 2) definitive hematopoiesis, beginning in the fetal liver and maintained in the BM throughout life. In a number of organs, resident-macrophage populations derived from primitive hematopoiesis have been shown to be maintained through adulthood in mice by local proliferation. In the heart, steady-state cardiac macrophages have been found through genetic fate mapping to primarily originate from the yolk-sac, maintained by local proliferation (Epelman et al., 2014; Heidt et al., 2014). However, following myocardial infarction (MI), these resident macrophages are replaced by an influx of monocytes that mature into both macrophages with inflammatory (M1) and tissue-repair (M2) phenotypes (Heidt et al., 2014; Hilgendorf et al., 2014).

In the vasculature, a population of Sca1^+^CD45^+^ adventitial macrophage progenitor cells (AMPCs) have been identified, which are proposed to be derived by local proliferation maintained from prenatal development and which are upregulated in atherosclerosis (Psaltis et al., 2014). Adoptive transfer of GFP^+^ AMPCs into the carotid artery of *Apoe* *^−/−^* mice prior to 16 weeks on a Western-type diet demonstrated that these AMPCs contributed to macrophage populations, primarily in the adventitia, but also to a lesser degree in the plaque. However, while this study suggested that the BM and spleen are not able to efficiently reconstitute AMPC populations, definitive fate mapping studies are required to determine whether these cells are indeed true resident tissue macrophages seeded prenatally. The Robbins group performed fate mapping studies showing the contribution of embryonic macrophage precursors and BM HSPCs within the aorta postnatally (Ensan et al., 2016). Through pulse-labeling of CX3CR1 and FLT3, this study identified that while both embryonic and BM-derived precursors maintain the aortic macrophage repertoire, respectively, BM HSPC contribution declines throughout adulthood, at least under homeostatic conditions. While this study demonstrates the interplay between two defined macrophage precursor populations in the steady state, further fate mapping studies during the development and progression of atherosclerosis, as well as in metabolic disease, are required to delineate the significance of the contribution of the resident macrophage pool vs. monocyte-derived macrophages.

## Local Macrophage Proliferation

The atherosclerotic lesion, in theory, requires a constant turnover of macrophages to promote the resolution of this inflammatory pathology of the vascular wall. Within the plaque, macrophages have been suggested to proliferate to maintain macrophage content, particularly within more advanced lesions. Robbins et al. (2013) performed elegant experiments utilizing parabiosis of CD45.1 and CD45.2 mice in combination with BrdU labeling to measure proliferating macrophages within the plaque. From this study, they concluded that, during early lesion development, only ∼30% of plaque macrophages were derived from local proliferation, with the remainder derived from monocytes; however, in advanced lesions, the contribution of macrophage proliferation increased to ∼87% of total plaque macrophage content. Importantly, however, in the same study, parabiosis experiments conducted over a few months revealed that all locally proliferating macrophages were replaced by recruited monocytes (as we have discussed previously) (Murphy and Tall, 2014). Perhaps plaque monocytes/macrophages undergo a round of proliferation in response to the environment but then either die in or leave the lesion, and hence, new blood-derived monocytes are recruited. This ultimately suggests that monocyte entry is the rate-limiting step in atherosclerotic lesion progression.

In the context of diabetes, Lamharzi et al. (2004) have demonstrated that diabetes-associated hyperlipidemia can exacerbate plaque macrophage proliferation, which appears to require the hyperlipidemic environment as this phenotype was not seen in non-hyperlipidemic diabetic mice. Further, while this study showed that atherosclerotic and hyperglycemic conditions stimulate proliferation *in vitro*, the *in vivo* findings were based on BrdU labeling with prior studies showing that BrdU is incorporated into monocytes, and thus monocyte-derived macrophages, as well as locally proliferating macrophages (Yona et al., 2013). Given the technical caveat of distinguishing BrdU^+^ staining between proliferating local macrophages and BM progenitors, of which monocyte-derived macrophages are derived, there are currently no studies that have conclusively determined the contribution of local macrophage proliferation in diabetes or obesity. Moreover, studies focused on local macrophage proliferation in atherosclerosis rely on macrophage markers that do not necessarily represent “true” (myeloid-derived) macrophages. Fate mapping with confetti mice (Wang et al., 2013) could be employed in atherosclerotic models to fate map clones of hematopoietic-derived monocytes/macrophages. Approaches such as this should help to more faithfully determine the contribution of recruited cells to the atherosclerotic plaque. In human lesions, very few proliferating cells are detected within the lesions by proliferating cell nuclear antigen (PCNA) staining (<1% in the majority of patient samples), with 27.1% of these PCNA^+^ cells identified as macrophages (HAM56^+^) but only 3.9% of these were Mac 387^+^ (Gordon et al., 1990). In addition, 15.5% of PCNA^+^ cells in human lesions were found to be HHF35^+^, indicating VSMCs. Importantly, however, PCNA staining only detects cell proliferation at an individual timepoint and may therefore underestimate the percentage of macrophages within the plaque that retain the capacity to proliferate locally.

## Vascular Smooth Muscle Cell-Derived Macrophage-Like Cells

More recently, VSMCs have been shown to transdifferentiate into macrophage-like cells in atherosclerotic plaques. The first evidence for this *in vitro* was provided by the Fisher group where they demonstrated that VSMCs have the capacity to take up and accumulate cholesterol to form foam cells (Rong et al., 2003). This process was associated with a decrease in smooth muscle cell surface markers and gene expression, which coincided with an upregulation of macrophage genes and surface markers including CD68 and Mac-2. However, despite the expression of macrophages markers, these macrophage-like cells have lower phagocytic and efferocytic capacity compared to activated peritoneal macrophages, and gene expression of these cells indicates that their phenotype remains closer to VSMCs than macrophages (Vengrenyuk et al., 2015).

*In vivo*, genetic inducible fate mapping of VSMCs demonstrated that, during atherosclerosis, VSMCs undergo clonal expansion to form the fibrous cap. Within 8 weeks of high-fat feeding, a large number of clonal VSMCs migrate to the plaque core where they undergo transdifferentiation, downregulating SMC markers and upregulating macrophage markers (Misra et al., 2018). While these cells only have approximately a fifth of the proliferative capacity of VSMCs in the cap, these VSMC-derived cells constitute the majority of proliferative cells in the core, indicating that VSMCs and macrophage-like cells could contribute largely to the previously described local “macrophage” proliferation. Transdifferentiation of the VSMC has been shown to require a reduction in integrin β3 (Itgβ3) to induce upregulation of toll-like receptor 4 (TLR4), resulting in an increase in CD36 and a cholesterol-induced phenotypic switch towards a more macrophage-like cell (Misra et al., 2018). Complementing *in vitro* studies, macrophage-like cells contain oxLDL and exhibit foam cell-like morphology *in vivo* (Feil et al., 2014; Misra et al., 2018). However, while in steady-state atherosclerotic models, VSMC transdifferentiation has been shown to contribute to the “macrophage” content of atherosclerotic plaques identified by traditional macrophage markers, the exact role of these macrophage-like cells in the pathogenesis of the disease remains to be determined. Moreover, the contribution of VSMC-derived macrophage-like cells to atherosclerotic plaques in diabetes and obesity have yet to be established. *In vitro*, hyperglycemia has been shown to induce VSMC proliferation, migration, and inflammatory gene expression suggestive of a potential to induce transdifferentiation *in vivo* (Qi et al., 2012).

## Monocyte Subsets in Atherosclerosis

In mice, monocytes can be divided into two major subsets: classical CCR2^hi^CX3CR1^lo^Ly6C^hi^ monocytes and non-classical CCR2^lo^CX3CR1^hi^Ly6C^lo^ monocytes. Fate mapping and adoptive transfer studies indicate that Ly6C^lo^ monocytes develop from the Ly6C^hi^ subset (Swirski et al., 2007; Yona et al., 2013). Hypercholesterolemia has been shown to expand the Ly6C^hi^ subset and has been proposed to impair their maturation towards Ly6C^lo^ monocytes (Swirski et al., 2007). Ly6C^hi^ monocytes are more inflammatory compared to Ly6C^lo^ monocytes and, in atherosclerosis, Ly6C^hi^ monocytes preferentially infiltrate the arterial wall (Swirski et al., 2007; Tacke et al., 2007; Potteaux et al., 2011). Specific depletion of Ly6C^hi^ monocytes reduced lesion progression and lowered macrophage and apoptotic cell content (Soehnlein et al., 2013). Importantly, CCR2 and CX3CR1 are both required for Ly6C^hi^ monocyte recruitment to plaques, while Ly6C^lo^ recruited monocytes, despite their higher expression of CX3CR1, are independent of these chemokine receptors (Tacke et al., 2007). Deletion of CX3CR1, CCL2, or its receptor, CCR2, has been shown to impede atherosclerotic progression, suggesting that recruitment of the Ly6C^hi^ monocyte subset is responsible for promoting atherogenesis (Kuziel et al., 2003; Combadiere et al., 2008). In contrast, deletion of CCR5, required for Ly6C^lo^, but not Ly6C^hi^, monocyte recruitment, has been shown not to effect atherosclerotic progression, at least in early lesions (Kuziel et al., 2003). However, in mice lacking CCL2 and CX3CR1, inhibition of CCR5 further reduces atherogenesis, indicating that Ly6C^lo^ monocytes contribute to atherogenesis in the absence of Ly6C^hi^ monocyte recruitment (Combadiere et al., 2008). Mice deficient in Nur77 (also known as NR4A1), a transcription factor that is shown to be required for Ly6C^lo^ monocyte differentiation and survival (Hanna et al., 2011), exhibit accelerated atherosclerosis in some studies but not in others (Hamers et al., 2012; Hanna et al., 2012; Chao et al., 2013). Interestingly, however, CCR2 and CX3CR1 have also been shown to be required for the resolution of inflammation and plaque regression, indicating that the recruitment of Ly6C^hi^ monocytes is required for plaque regression (Rahman et al., 2017). However, in the context of diabetes, we and others have previously demonstrated increased Ly6C^hi^ monocyte entry in regressing plaques, associated with increased inflammatory gene expression and reduced M2 polarization, which could suggest that diabetes impairs Ly6C^hi^ monocyte differentiation to M2 macrophages and/or that excess Ly6-C^hi^ monocytes may also play a deleterious role in plaque regression (Parathath et al., 2011; Nagareddy et al., 2013).

In humans, monocyte subsets are defined based on their CD14 and CD16 expression, with classical monocytes (CD14^++^CD16^−^) traditionally recognized as being analogous to the Ly6C^hi^ subset and non-classical monocytes (CD14^dim^CD16^+^) being equivalent to the Ly6C^lo^ subset in mice. Fate mapping in human monocytes has demonstrated that CD14^dim^CD16^+^ monocytes develop from CD14^++^CD16^−^ monocytes by transitioning through a third population of CD14^+^CD16^+^ intermediate monocytes (Korkosz et al., 2012). All three populations are found in the circulation with classical monocytes comprising 80–90% of total circulating monocytes under homeostatic conditions, while non-classical and intermediate monocytes comprise 2–10% and 2–5%, respectively. In contrast to the role of Ly6C^hi^ monocytes in driving atherosclerotic progression in mice, evidence largely supports a role for CD16^+^ monocytes including non-classical monocytes, as well as intermediate monocytes, in increasing CVD risk (Schlitt et al., 2004; Hristov et al., 2010; Rogacev et al., 2010; Rogacev et al., 2011; Rogacev et al., 2014). *In vitro*, all human monocyte subsets polarize to M1 macrophage in response to LPS/IFNγ or GM-CSF or to M2 macrophages in response to M-CSF (Al-Sharea et al., 2016; Boyette et al., 2017). In contrast, IL-4 also induces M2 polarization in classical and intermediate but not non-classical monocytes (Al-Sharea et al., 2016). Importantly, both M1 and M2 macrophage function has been shown to differ based on the monocyte subset from which they were derived, with those from classical monocytes exhibiting higher phagocytic capacity than that of intermediate or non-classical monocyte-derived macrophages (Boyette et al., 2017).

Further advances in immune cell profiling with the introduction of CyTOF mass cytometry also indicate that the use of additional markers in defining these subsets may help to improve the potential of monocyte subsets as predictive determinants of cardiovascular risk (Thomas et al., 2017). However, although circulating levels of these monocyte subsets are associated with CVD, it remains unknown what capacity these monocyte subsets have to infiltrate the plaque and promote atherosclerosis. The development of new humanized immune system mouse models of atherosclerosis that support all the monocyte subsets could allow for the direct study of human monocyte subsets in the plaque to determine whether these subsets play a causal role in promoting atherogenesis or plaque instability.

## Monocyte Subsets in Diabetes and Obesity

Diabetes and obesity are associated with increased circulating monocytes (Nagareddy et al., 2013; Nagareddy et al., 2014). In diabetic mice, this is predominantly driven by an increase in the Ly6C^hi^ subset, whereas diet-induced obesity (DIO) results in an expansion of both Ly6C^hi^ and Ly6C^lo^ monocytes (Nagareddy et al., 2013; Nagareddy et al., 2014). It has previously been suggested that hypercholesterolemia may act to inhibit the conversion of Ly6C^hi^ to Ly6C^lo^ monocytes (Swirski et al., 2007), which, given the hypercholesterolemia present in diabetic mouse models, could explain the difference in monocyte phenotypes between diabetes and DIO.

In humans, obesity, measured either as fat mass or by the WHO obesity classification, has been shown to be associated with an increase in intermediate (CD14^+^CD16^+^) and non-classical (CD14^dim^CD16^+^) monocytes (Stansfield and Ingram, 2015). Obese patients with diabetes exhibit an even greater increase in non-classical monocytes but with similar levels of intermediate monocytes compared to obese non-diabetic patients (Poitou et al., 2011). Roux-en-Y gastric bypass surgery—which is known to reduce cardiovascular risk in obese patients with diabetes and is associated with reductions in weight loss and improved glycemic control—significantly reduced both monocyte subsets (Poitou et al., 2011). Moreover, this decrease in the CD14^+^CD16^+^ monocyte subset is associated with decreased intima-media thickness, suggesting that the intermediate monocyte subset may contribute to accelerated atherosclerosis and increased risk of CVD in diabetes and obesity. Similar effects on CD14^+^CD16^+^ monocytes were also observed in morbidly obese patients following gastric bypass with no difference observed between diabetic and non-diabetic individuals (Cottam et al., 2002).

## Myeloid-Derived Suppressor Cells

Myeloid-derived suppressor cells (MDSCs) are a population of immune regulatory myeloid cells that have been identified in a number of pathological conditions, most notably in cancer, but also in metabolic disease. MDSCs are defined as either monocytic MDSCs (mMDSCs, low side scatter CD11b^+^Ly6G^-^Ly6C^hi^) or granulocytic MDSCs (gMDSCs, high side scatter CD11b^+^Ly6G^+^Ly6C^lo^). As such, mMDSCs share the same markers as Ly6Chi inflammatory monocytes; however, they typically lack the monocytic markers CD11c and MHC class II. These MDSC are known to develop from monocyte and neutrophil precursors in the bone marrow, where a number of cytokines, lipid mediators, and growth factors have been shown to reprogram development toward MDCS. In humans, mMDSCs are classified as CD14^+^HLA-DR^-/lo^, in contrast to the monocyte subsets, which are HLA-DR^+^, and are increased in the blood of patients with acute coronary syndrome (ACS) (Wang et al., 2015). However, despite this association, there is little direct evidence for a role of mMDSCs in atherosclerosis. In an attempt to assess the role of MDSCs in atherosclerosis, a study by Foks et al. (2016) utilized repetitive adoptive transfer of bone marrow CD11b^+^Gr1^+^ cells, from *Ldlr* *^−/−^* mice fed a WTD for 2 weeks and deemed to be MDSCs, into *Ldlr* *^−/−^* mice on a WTD for 6 weeks, demonstrating that these cell transfers reduce lesion size. Importantly, however, although the co-culture of these cells with T cells demonstrated suppressive features of MDSCs, the definition of MDSCs in this study does not exclude classical neutrophils or Ly6C^hi^ monocytes, which comprise a large portion of the bone marrow. Moreover, while these cells were tracked to spleen, lymph nodes, and adipose tissue, it was not determined whether these cells could traffic to the plaque. Moreover, while adoptive transfer studies provide a foundation indicating an antiatherogenic role for these cells, the artificial nature of these experiments makes it difficult to assess whether these cells would naturally maintain these immunosuppressive characteristics during normal or pathological trafficking from the bone marrow. Further studies, delineating mMDSCs from Ly6C^hi^ monocytes, using additional markers and lineage tracing, are required to directly track the contribution and causal role of these cells in the development of atherosclerosis. In humans, MDSCs increased the circulation of obese patients and patients with T1D (Whitfield-Larry et al., 2014; Bao et al., 2015). In obese (high-fat-fed ob/ob) mice, CD11b^+^GR1^+^ have been found to accumulate in the spleen, liver, and epididymal fat (Xia et al., 2011). Whether obesity or diabetes promotes the accumulation of MDSCs in the plaque remains to be seen. More importantly, while MDSCs may play an anti-atherogenic role, their potential contribution in metabolic disease does not appear to outweigh the pro-atherogenic role of macrophages within the lesion.

## Myelopoiesis in Metabolic and Inflammatory Disease

Monocytes originate in the bone marrow where they develop from hematopoietic stem cells through a process called myelopoiesis. Myelopoiesis is a subbranch of hematopoiesis specifically involving the production of myeloid cells, which include monocytes as well as dendritic cells, granulocytes, platelets, and red blood cells. Hematopoietic stem cell commitment towards the monocytic lineage involves the progressive differentiation through a number of myeloid and monocyte precursors including, in order from least to most committed, the common myeloid progenitor (CMP), granulocyte–macrophage progenitor (GMP), monocyte–dendritic cell progenitor (MDP), and common monocyte progenitor (cMOP) (Hettinger et al., 2013). Enhanced myelopoiesis is known to result in monocytosis and accelerates the progression of atherosclerosis, as well as impairing atherosclerotic regression (Swirski et al., 2007; Tacke et al., 2007; Murphy et al., 2011; Nagareddy et al., 2013; Distel et al., 2014). Furthermore, under inflammatory settings, hematopoietic stem and progenitor cells (HSPCs) can mobilize to the spleen where they give rise to additional monocytes through extramedullary myelopoiesis (Robbins et al., 2012).

Myelopoiesis can be modulated by a number of metabolic and inflammatory diseases associated with an increased risk of CVD including hypercholesterolemia, rheumatoid arthritis (RA), MI, as well as both diabetes and obesity. In hypercholesterolemia, myelopoiesis is enhanced by cholesterol loading of stem cells, which increases proliferation of these cells, resulting in augmented monocyte production (Swirski et al., 2007; Tacke et al., 2007; Yvan-Charvet et al., 2010; Murphy et al., 2011). Hypercholesterolemia is also associated with stem cell mobilization and extramedullary myelopoiesis, giving rise to Ly6C^hi^ monocytes, which accelerate atherosclerosis (Robbins et al., 2012). In RA, myelopoiesis is induced *via* a similar mechanism whereby inflammatory signals impair cholesterol efflux mechanisms, resulting in a proliferative stem cell phenotype and extramedullary myelopoiesis (Dragoljevic et al., 2018). Following MI, monocytes are mobilized to the heart with Ly6C^hi^-driven monocytosis, resulting in increased Ly6C^hi^ accumulation within the first 4 days post-MI, while Ly6C^lo^ monocytes were shown to preferentially accumulate 5–7 days post-MI following changes in chemotactic signals (Nahrendorf et al., 2007). MI-induced β3-adrenergic signaling induces the release of stem cells from the BM, following which they migrate to and seed the spleen. The spleen becomes a major organ for extramedullary myelopoiesis, contributing to an already elevated pool of circulating Ly6C^hi^ monocytes that would readily enter the inflamed arteries to accelerate atherosclerosis (Dutta et al., 2012). In contrast, diabetic (both STZ and Akita models) and obese (DIO, db/db and ob/ob) mice exhibit enhanced myelopoiesis within the BM (i.e., medullary), but not extramedullary, suggesting a separate signaling pathway for myelopoiesis in these metabolic diseases (Nagareddy et al., 2013; Nagareddy et al., 2014).

## S100A8/A9 as a Mediator of Myelopoiesis in Metabolic Disease

**Figure 2:**
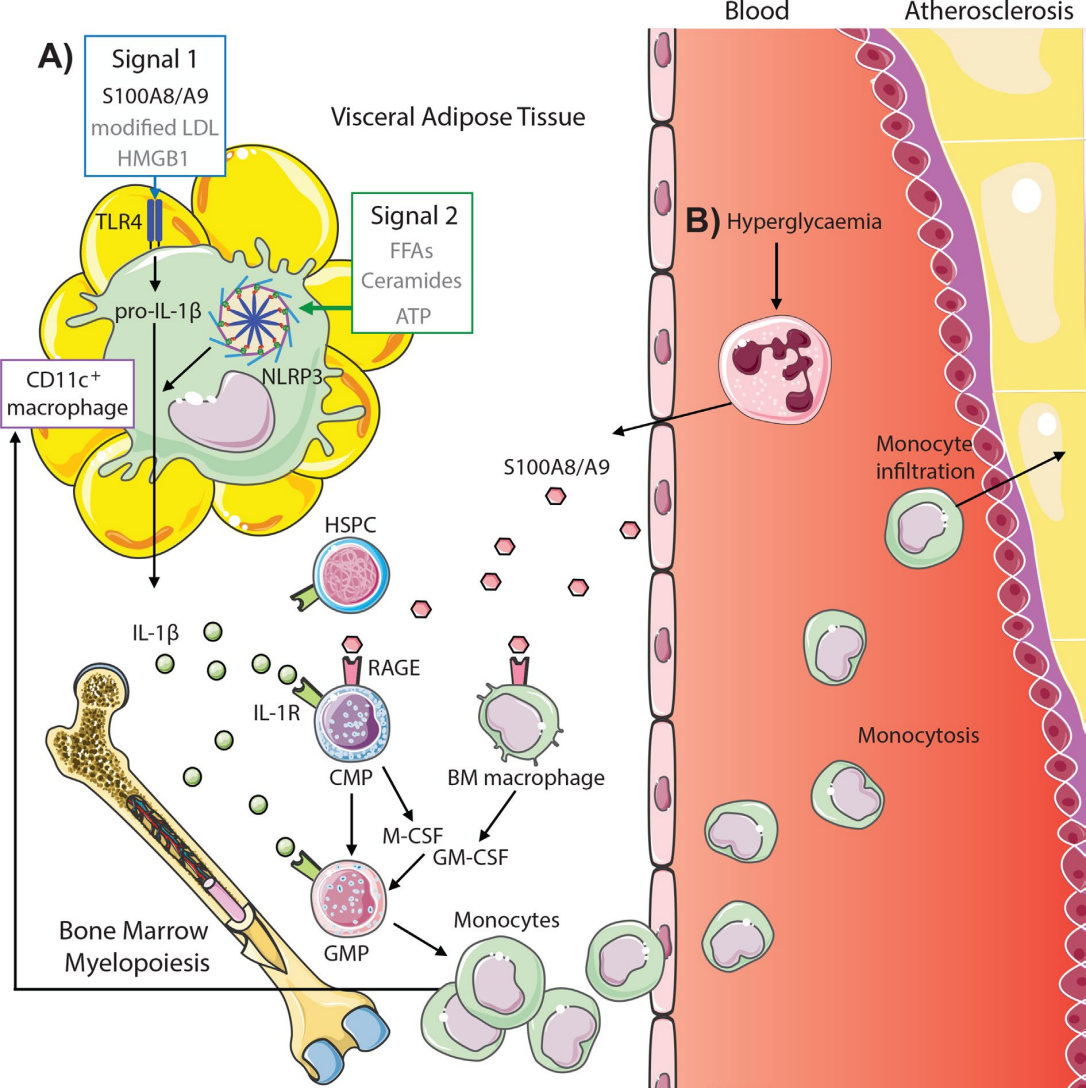
S100A8/A9 drives myelopoiesis and monocytosis in diabetes and obesity. **(A)** Adipose tissue inflammation in obesity promotes monocytosis through S100A8/A9-TLR4 signaling on CD11c^+^ adipose tissue macrophages (signal 1, other potential mediators in gray) and activation of the NLRP3 inflammasome (signal 2) to promote IL-1β, which signals through the IL1R on common myeloid progenitors (CMPs) and granulocyte–macrophage progenitors (GMPs) to induce myelopoiesis. This results in both increased circulating monocytes and feeds back to increase CD11c^+^ macrophages. **(B)** Hyperglycemia promotes monocytosis by direct signaling of neutrophil-derived S100A8/A9 *via* RAGE on CMPs and macrophages in the bone marrow, promoting CMP and GMP proliferation and differentiation *via* autocrine and paracrine (M-CSF and GM-CSF) signaling, respectively. These monocytes infiltrate atherosclerotic lesions to promote atherogenesis (further detailed in **Figure 1**).

S100A8/A9, a damage-associated molecular pattern protein heterodimer, correlates with the severity of coronary artery disease in diabetic patients as well as with HbA1c levels and body mass index (BMI) (Peng et al., 2011; Nagareddy et al., 2013; Cotoi et al., 2014). Moreover, neutrophils, the primary source of S100A8/A9, are likewise associated with both coronary events and cardiovascular deaths (Cotoi et al., 2014). We have previously shown that S100A8/A9 is responsible for inducing myelopoiesis in both diabetes and obesity; however, the mechanism by which S100A8/A9 mediates this process differs between disease contexts, which we discuss below (**Figure 2**).

## Myelopoiesis in Diabetes

In diabetes, we found that monocytosis is consequential of hyperglycemia-induced upregulation of plasma S100A8/A9 (Nagareddy et al., 2013). In this setting, S100A8/A9 was shown to interact directly with RAGE on CMPs and macrophages in the BM, activating NF-κB signaling within these cells to promote the secretion of M-CSF and GM-CSF, respectively. GM-CSF and M-CSF promote the proliferation and differentiation of GMPs, with M-CSF directing proliferation of CMPs *via* autocrine signaling. S100A8/A9 also directly induces the proliferation CD34^+^ stem cell progenitors from human cord blood *in vitro*. This is likely due to signaling *via* RAGE on CMPs and results in increased CD14^+^ monocytes. Moreover, controlling glucose levels in diabetic mice with a sodium glucose co-transporter 2 inhibitor (SGLT2i) reduces circulating S100A8/A9 and prevents both diabetes-induced myelopoiesis and atherosclerosis. In addition to promoting monocytosis, hyperglycemia-induced S100A8/A9 also results in increased production of highly reactive platelets (through an independent mechanism), which together results in increased platelet–leukocyte interactions (Kraakman et al., 2017). Again, inhibiting S100A8/A9 is effective in dampening this process.

Hyperglycemia also promotes the formation of advanced glycation end products (AGEs), as well as carbonyl intermediates such as glycoaldehyde (GA), glyoxal (GO), and methylglyoxal (MGO), which are elevated in diabetes. Carbonyls (specifically GA and GO) have been shown to destabilize ABCA1 and impair cholesterol efflux in macrophages *in vitro* (Passarelli et al., 2005). ABCA1 upregulation in diabetic mice *via* treatment with the anti-miR33 prevents diabetes-induced myelopoiesis and monocytosis, but not neutrophilia (Distel et al., 2014). Anti-miR33 treatment also prevented diabetes-induced monocyte recruitment and improved M2-switching and macrophage reduction during atherosclerotic regression (Distel et al., 2014). This was likely mediated *via* the reduction in myelopoiesis through stabilization of ABCA1 and HDL-mediated cholesterol transport in CMPs and GMPs as well as the direct upregulation of ABCA1 observed in plaque macrophages, improving cholesterol efferocytosis within the plaque. While plasma HDL-C was increased by anti-miR33 treatment, total cholesterol, triglycerides, and plasma glucose levels were increased in diabetes but not altered by anti-miR33 treatment. Blocking ABCA1 has also been shown to prevent the anti-inflammatory effects of apolipoprotein A-I (apoA-I) from HDL on monocytes, indicating that carbonyl-mediated destabilization of ABCA1 likely impairs effects of HDL on macrophages, CMPs, and GMPs (Murphy et al., 2008). Plasma MGO levels have also been shown to be associated with increased incidence of CVD in patients with type 1 and type 2 diabetes (Hanssen et al., 2017; Hanssen et al., 2018); however, whether MGO influences cholesterol handling in stem or progenitor cells and influences myelopoiesis remains unknown.

Monocyte levels have also been shown to be associated with plasma norepinephrine in diabetic patients, which has been proposed to modulate extramedullary myelopoiesis (Vasamsetti et al., 2018). In contrast with previous studies that showed no change in GMPs in the spleen (Nagareddy et al., 2013), this study showed increased splenic GMPs in diabetes, which were more proliferative and were shown *via* adoptive transfer experiments to differentiate more readily, generating higher numbers of myeloid cells. Importantly, however, GMPs in this study were gated more strictly, resulting in a much smaller population containing only the most CD34^hi^ subset, which may suggest that further heterogeneity exists within the classically defined GMP population than previously thought. How the GMPs arrived in the spleen is unknown given that these cells have an ∼2-week half-life in the spleens of atherogenic mice (Robbins et al., 2012), and it is well known that HSPC mobilization from the BM is severely perturbed in diabetes (Ferraro et al., 2011). However, it should be noted that Vasamsetti *et al.* did reveal that ablation of sympathetic signaling in the spleen did reduce GMP proliferation. Whether plasma norepinephrine influences the proliferative or differentiative potential of other myeloid progenitors remains unknown.

## Myelopoiesis in Obesity

Interestingly, in models of DIO, we found that S100A8/A9 did not significantly increase in the plasma but were instead elevated locally within the visceral adipose tissue (VAT) (Nagareddy et al., 2014). This reflected measurements in the plasma and adipose tissue from lean and obese humans. Here, S100A8/A9 signaled through TLR4 on CD11c^+^ adipose tissue macrophages (ATMs) to promote the production of IL-1β, acting as a myeloproliferative signal in the bone marrow, thereby inducing monocytosis. Alongside S100A8/A9, a number of endogenous TLR-4 ligands including gut-derived LPS, HMGB1, and modified LDL are elevated in the plasma and adipose tissue in obesity and may contribute to ATM priming to produce IL-1β (Erridge, 2010). Obesity is also associated with elevated levels of free fatty acids (FFAs), which were originally postulated to act as a TLR4 agonist, as TLR4*^−/−^* mice are protected from obesity-associated inflammation (Davis et al., 2008; Osborn and Olefsky, 2012). However, recent evidence suggests that FFAs such as palmitate promote macrophage inflammation, a process that requires TLR4 signaling, but do not bind to TLR4 directly (Lancaster et al., 2018). TLR4 signaling induces pro-IL-1β production, which requires cleavage by the NLRP3 inflammasome to produce functional IL-1β, and as such, secondary signals are needed to activate the NLRP3 inflammasome (Sims and Smith, 2010). In obesity, FFAs as well as ceramides and extracellular ATP have all been suggested to activate NLRP3 (Vandanmagsar et al., 2011; Wen et al., 2011; Moon et al., 2016). Together, this suggests that, while S100A8/A9 plays an important role in inducing IL-1β production, additional inflammatory signals within the adipose tissue likely co-signal to promote IL-1β and myelopoiesis in obesity.

## Trained Immunity in Metabolic Disease

Given that metabolic diseases are chronic, yet have the ability to be reversed through changes in diet and exercise leading to whole-body metabolism improvement and weight loss, it is important to know the length of the effects on the hematopoietic system. Through serial bone marrow transplants, hematopoietic stem cells from obese mice have been shown to retain an increased potential to produce myeloid cells compared to those originating from lean mice (Singer et al., 2014). These cells produced more CD11c^+^ BM cells and overall increase in both total and CD11c^+^ ATMs once re-exposed to DIO. These results suggest that potential epigenetic regulation of stem cells in obesity could promote myeloid skewing. This production of CD11c^+^ ATMs could also feed-forward to enhance the IL-1β signaling induced by S100A8/A9 in obesity to promote myelopoiesis and monocytosis. A recent study by the Latz group demonstrated that a Western-type diet induces both transcriptomic and epigenomic reprogramming in GMPs towards the “trained immunity” phenotype, mediated *via* NLRP3 signaling (Christ et al., 2018). In β-glucan models of trained immunity, IL-1β acts on HSPCs to modulate myelopoiesis (Mitroulis et al., 2018). It is therefore likely that IL-1β signaling from ATMs could also potentiate trained immunity within the context of obesity and that S100A8/A9 may therefore play a role in trained immunity upstream of IL-1β.

Likewise, in diabetes, it is postulated that hyperglycemia induces long-term effects on the immune system through a process known as hyperglycemic memory, mediated through epigenetic modifications. Supporting this, clinical studies have demonstrated that improved glucose control has sustained effects following the return to usual glucose control (Nathan and Group, 2014). Furthermore, in recent years, it has become apparent that diabetic as well as pre-diabetic patients exhibit severe variations in glycemia, and post-prandial hyperglycemia is an independent risk factor for CVD (Decode Study Group, 2001; Ning et al., 2012; Monnier and Colette, 2015; Hall et al., 2018). Pre-clinically, transient hyperglycemia has been shown to induce epigenetic changes in the vasculature, which was associated with increased NFκB signaling (Brasacchio et al., 2009). It would therefore be interesting to determine whether hyperglycemia also results in trained immunity by inducing epigenetic changes within the stem and progenitor cells in the BM. Moreover, given the presence of S100A8/A9-driven myelopoiesis in the setting of chronic hyperglycemia, it would also be interesting to determine whether fluctuations in glycemia, in particular transient hyperglycemia, could promote S100A8/A9 signaling to induce myelopoiesis and whether this could have downstream effects on atherogenesis.

## Effects of Weight Loss on S100A8/A9 and Monocytosis

Despite strong evidence that obesity induces trained immunity, we have previously shown that weight loss reduces monocyte and neutrophil levels in diet-induced obese mice, suggesting that preventing obesity-related inflammatory signals is still effective in reducing myelopoiesis (Nagareddy et al., 2014). Likewise, monocyte and neutrophil levels were also found to be significantly reduced in obese patients following bariatric surgery associated with an average weight loss of 26.7% (Nagareddy et al., 2014). Moreover, a recent study in obese patients with or without type 2 diabetes mellitus (T2DM) showed that plasma S100A8/A9 was significantly reduced following Roux-en-Y gastric bypass surgery independent of diabetes (Lylloff et al., 2017). However, it is important to note that while non-diabetic patients were classified as having Hba1c below 6.5% (48 mmol/mol), Hba1c levels decreased in all groups. Unfortunately, independent associations of plasma S100A8/A9 with Hba1c or BMI were not reported. While monocytes were not specifically measured in this study, total leukocytes were associated with S100A8/A9 levels and were reduced and in obese/diabetic patients following surgery. Together, these data suggest that weight loss reduces S100A8/A9 and monocytosis in humans; however, whether this is a consequence of reduced adiposity, improved glucose tolerance, or both is yet to be delineated.

## S100A8/A9 as a Potential Therapeutic Target in Metabolic Disease-Related CVD

Given the role of S100A8/A9 in atherosclerosis in both diabetes and obesity-associated myelopoiesis and the subsequent effects of monocytosis on atherosclerosis, targeting S100A8/A9 could be a potential therapeutic target to treat monocytosis and atherosclerosis in metabolic disease. Indeed, we have previously shown that blocking S100A8/A9 signaling, using a small molecular inhibitor ABR-215757 (Paquinimod), in STZ-diabetic mice prevents hyperglycemia-induced atherogenesis and reduces plaque macrophage content (Kraakman et al., 2017). Although it has yet to be explored whether S100A8/A9 promotes myelopoiesis in the context of other metabolic and cardiovascular risk factors, plasma levels of S100A8/A9 are also associated with hyperlipidemia and smoking and are increased following MI, which suggests that S100A8/A9 may be, at least in part, responsible for this increase in circulating myeloid cells and could therefore contribute to plaque macrophage accumulation and atherosclerosis in these patients (Du et al., 2012; Schiopu and Cotoi, 2013).

## Conclusions and Future Directions

Diabetes and obesity are associated with exacerbated macrophage inflammation, which contributes to an overall increase in cardiovascular risk. Macrophages play a crucial role in the development of CVD by promoting atherosclerosis and contribute to plaque vulnerability. Macrophages within the plaque are a heterogenous population known to be derived from a number of sources, consisting of both true macrophages and macrophage-like cells, which may contribute differently to lesion development or regression. Diabetes and obesity are known to contribute to macrophage accumulation by enhancing myelopoiesis to increase circulating and infiltrating monocytes; however, metabolic abnormalities such as hyperglycemia and hypercholesterolemia may also contribute to macrophage accumulation by influencing the development and proliferation of alternative sources of macrophages. The contributions of locally produced macrophages and monocyte-derived macrophages in promoting macrophage accumulation and promoting atherosclerosis requires further study, particularly in the context of metabolic disease. Targeting the production of monocyte-derived macrophages has proven to be effective in reducing atherosclerosis pre-clinically in a number of metabolic and inflammatory diseases including hypercholesterolemia, RA, obesity, and diabetes. In particular, S100A8/A9 has emerged as a key myeloproliferative factor in diabetes and obesity, and as such, treatment with inhibitors of S100A8/A9 in addition to current therapies may reduce CVD risk in these patient groups.

## Author Contributions

All authors contributed to writing and drafting the review.

## Funding

AM was supported by a Centenary Award from CSL. PN was supported by grants from the NIH (R01HL1379 and R00HL1225).

## Conflict of Interest Statement

The authors declare that the research was conducted in the absence of any commercial or financial relationships that could be construed as a potential conflict of interest.
